# Shoot Cultures of *Vitis vinifera* (Vine Grape) Different Cultivars as a Promising Innovative Cosmetic Raw Material—Phytochemical Profiling, Antioxidant Potential, and Whitening Activity

**DOI:** 10.3390/molecules28196868

**Published:** 2023-09-29

**Authors:** Marta Sharafan, Magdalena Anna Malinowska, Marta Kubicz, Paweł Kubica, Marin-Pierre Gémin, Cécile Abdallah, Manon Ferrier, Christophe Hano, Nathalie Giglioli-Guivarc’h, Elżbieta Sikora, Arnaud Lanoue, Agnieszka Szopa

**Affiliations:** 1 Department of Pharmaceutical Botany, Medical College, Jagiellonian University, Medyczna 9 St., 30-688 Cracow, Poland; marta.sharafan@doktorant.pk.edu.pl (M.S.); marta.kubicz@student.uj.edu.pl (M.K.); p.kubica@uj.edu.pl (P.K.); 2 Institute of Organic Chemistry and Technology, Faculty of Chemical Engineering and Technology, Cracow University of Technology, 24 Warszawska St., 31-155 Cracow, Poland; elzbieta.sikora@pk.edu.pl; 3 EA 2106 Biomolecules et Biotechnologies Végétales, UFR des Sciences Pharmaceutiques, Université de Tours, 31 av. Monge, F37200 Tours, France; marin-pierre.gemin@univ-tours.fr (M.-P.G.); cecile.abdallah@ird.fr (C.A.); manon.ferrier@univ-tours.fr (M.F.); nathalie.guivarch@univ-tours.fr (N.G.-G.); arnaud.lanoue@univ-tours.fr (A.L.); 4 Institut de Chimie Organique et Analytique, Universite d’Orleans-CNRS, UMR 7311 BP 6759, CEDEX 2, 45067 Orléans, France

**Keywords:** *Vitis vinifera*, vine grape, in vitro culture, tissue culture, metabolomic profiling, stilbenoids, phenolic compounds, antioxidant capacity, anti-tyrosinase activity

## Abstract

The primary purpose of this work was the initiation and optimization of shoot cultures of different *Vitis vinifera* L. cultivars: cv. Chardonnay, cv. Hibernal, cv. Riesling, cv. Johanniter, cv. Solaris, cv. Cabernet Cortis, and cv. Regent. Cultures were maintained on 30-day growth cycles using two media, Murashige and Skoog (MS) and Schenk and Hildebrandt (SH), with various concentrations of plant growth regulators. Tested media (‘W1’–‘W4’) contained varying concentrations of 6-benzylaminopurine (BA) in addition to indole-3-butyric acid (IBA) and 1-naphthaleneacetic acid (NAA). High performance liquid chromatography coupled with mass spectrometry (UPLC–MS) was used for metabolomic profiling. In all tested extracts, 45 compounds were identified (6 amino acids, 4 phenolic acids, 13 flavan-3-ols, 3 flavonols, and 19 stilbenoids). Principal component analysis (PCA) was performed to assess the influence of the genotype and medium on metabolic content. PCA showed that metabolic content was mainly influenced by genotype and to a lesser extent by medium composition. MS media variants induced the amino acid, procyanidin, and flavan-3-ol production. In addition, the antioxidant potential and anti-tyrosinase activity was measured spectrophotometrically. The studies on antioxidant activity clearly reveal very high efficiency in reducing free radicals in the tested extracts. The strongest tyrosinase inhibition capacity was proved for shoots cv. Hibernal cultured in SH medium and supplemented with NAA, with an inhibition of 17.50%. These studies show that in vitro cultures of *V. vinifera* cvs. can be proposed as an alternative source of plant material that can be potentially used in cosmetic industry.

## 1. Introduction

*Vitis vinifera* L.—vine grape (Vitaceae) is a well-known species found in Europe (France, Italy, Spain), Asia (China) and the Americas (United States, Argentina, Chile) [1]. The raw materials obtained from the plant (fruit, seed, leaf, stem, cane, root) are widely used in food, pharmaceutical, and cosmetic industries. There are monographs published by respected organizations such as the EMA-HMPC (European Medicines Agency Committee on Herbal Medical Products) [2], FDA (Food and Drug Administration) [3], and EFSA (European Food and Safety Authority) [4] on the applicability of *V. vinifera* raw materials. In addition, there are nine *V. vinifera* raw materials, which can be used in cosmetics, that are listed in CosIng (Cosmetics Ingredient) database, namely: fruit, skin (peel), seed, leaf, flower, root, shoot, stem, and bud [5]. The extracts obtained from *V. vinifera* possess strong biological activity confirmed in numerous fields of scientific publications, including oncology, cardiology, hepatology, and neurology [6,7,8,9]. Moreover, *V. vinifera* extracts exhibit antioxidant, antimicrobial, anti-inflammatory, and skin-whitening activities, which are important in cosmetology [10,11,12,13,14,15,16,17,18]. The biological activity of *V. vinifera* can be attributed to its rich phytochemical composition [1,19,20]. The grape varieties that were the subjects of this research belong to the so-called PIWI varieties, collected in Jura region (South Poland), that are resistant to pathogens and cold climate. Their intricate composition of metabolites plays a pivotal role in conferring such resilience. These metabolites belong a diverse array of chemical classes such as phenolic compounds, flavonoids, and stilbenoids. These natural phytoalexins, notably resveratrol and quercetin, are known for their potent beneficial properties, which enhance the plant’s defense mechanisms against pathogens [21]. Furthermore, these compounds contribute to the reinforcement of the plant cell wall, inhibiting the ingress of pathogens. Flavonoids, such as anthocyanins and flavonols, provide protection by acting as UV-absorbing pigments and antimicrobial agents and contribute to the synthesis of essential oils, deterring pests and pathogens. The orchestrated interplay of these metabolites within the genetic makeup of the Polish grapevine cultivars play a role in pathogen defense and adaptability to cold climate, fostering the sustainable growth of viticulture in the region [22,23].

The secondary metabolites of *V. vinifera* which are responsible for biological activities, including antioxidant properties, are: phenolic acids (e.g., gallic acid, caffeic acid), flavonols (e.g., quercetin), flavan-3-ols (e.g., catechin), and stilbenoids (e.g., *trans*-resveratrol and its derivatives) [12,24,25]. *V. vinifera* stilbenoids (e.g., oxyresveratrol, *trans*-ε-viniferin) show skin-whitening properties [18,20,26,27,28]. The qualitative and quantitative differences of the phenolic constituents depend on the plant organ and grapevine variety, extraction technique, and environmental conditions [1,29,30,31,32].

Biotechnological methods create new, innovative possibilities for harvesting raw plant material. In vitro cultures are a highly appreciated source of biologically active compounds and increasingly used in cosmetology and the food and pharmaceutical industries. Plant biotechnology creates a wide range of possibilities for the cultivation of various species as well as different cultivars of a given species and the possibility of stimulating the production of biologically active compounds in the cultivated biomass [33,34,35,36,37,38].

The high utility of *V. vinifera* and the possibilities offered by plant biotechnology have made in vitro cultivars (cvs.) of grapevine a highly attractive target for natural cosmetics. The objects of our study were seven different in vitro shoot cultures of *V. vinifera*: five white varieties, Chardonnay, Hibernal, Riesling, Johanniter, and Solaris; and two red, Cabernet Cortis and Regent. The influence of Murashige and Skoog (MS) and Schenk and Hildebrandt (SH) agar media supplemented with different plant growth regulators (PGRs) were evaluated for their effects on the phytochemical composition and biological activities of generated biomass. The metabolomic profiling was performed using high performance liquid chromatography coupled with mass spectrometry (UPLC–MS). In addition, we evaluated the antioxidant and tyrosinase inhibitory activities of the biomass extracts.

## 2. Results

### 2.1. Appearance and Biomass Output of Shoot Cultures

**Figure 1:**
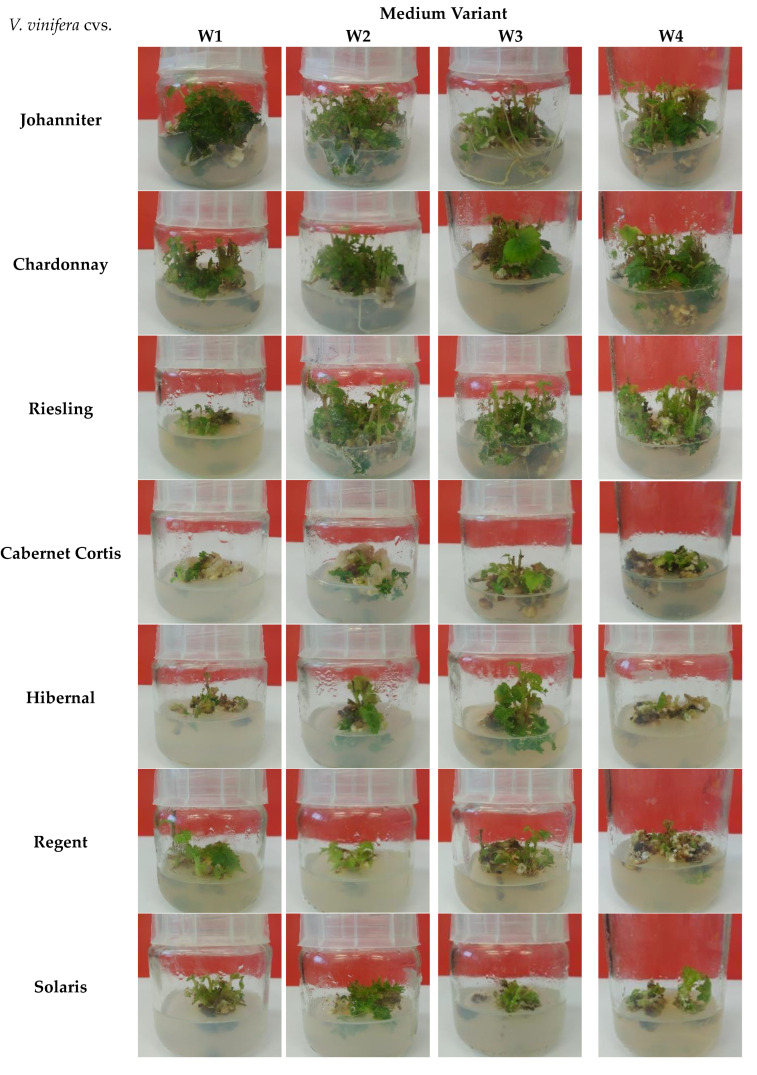
Growth of shoot cultures of various *V. vinifera* cvs. in differing variations of culture media (‘W1’—MS, 0.9 mg/L BA and 0.1 mg/L IBA, ‘W2’—MS, 1.5 mg/L BA and 0.2 mg/L NAA, ‘W3’—SH, 0.9 mg/L BA and 0.1 mg/L IBA, ‘W4’—SH, 1.5 mg/L BA and 0.2 mg/L NAA).

Cultures of experimental *V. vinifera* cvs. were carried out over 30-day periods (Figure 1). Biomass growth of *V. vinifera* cvs. was measured using Gi (growth index) factor (Table 1).

The cultures of *V. vinifera* cv. Johanniter in the ‘W3’ variant developed a root system, and the shoots were of a dark green color (Figure 1). However, the highest Gi value was found for cultures maintained in the ‘W2’ medium (Gi = 91.83) (Table 1).

*V. vinifera* cv. Chardonnay showed satisfactory growth and viability with each of the media variants. Numerous dark green shoots and leaves developed, and the formation of roots was observed in the ‘W2’ variant (Figure 1). The highest Gi value was seen for cultures maintained in the ‘W2’ variant (Gi = 95.89) (Table 1).

In vitro cultures of *V. vinifera* cv. Riesling showed good viability and growth in ‘W2’, ‘W3’, and ‘W4’ media variants. In ‘W1’, shoots and leaves were small and few (Figure 1). The highest Gi value was recorded for cultures grown in the ‘W3’ medium variant (Gi = 95.26) (Table 1).

For *V. vinifera* cv. Cabernet Cortis, after 30 days of cultivation, a significant increase in callus tissue was observed around shoots and stems in the ‘W1’ and ‘W2’ medium variants. In each of the media variants, the viability of the cultures was impaired (except for the ‘W3’ variant, showing slightly better growth) (Figure 1). The highest Gi value was observed for the cultures maintained in the ‘W2’ medium (Gi = 92.24) (Table 1).

For the *V. vinifera* cv. Hibernal, good growth and viability were observed for cultures grown in the ‘W2’ and ‘W3’ media variants. The cultures grown in ‘W1’ and ‘W4’ were characterized by significantly lower viability. Growth of leaves and shoots in ‘W2’ medium was the best. In variants ‘W2’ and ‘W3’, the shoots were dark-green, while in variants ‘W1’ and ‘W4’, they were yellow-green (Figure 1). The highest Gi value was found for cultures grown in ‘W2’ (Gi = 90.94) (Table 1).

In vitro cultures of *V. vinifera* cv. Regent after 30 days of cultivation were characterized by good viability in the ‘W1’ and ‘W2’ media variants. The ‘W3’ and ‘W4’ shoot viability was weaker with intensive development of callus tissue. Culture growth was the highest in ‘W1’ medium (Figure 1). The highest Gi value was found for cultures maintained in ‘W4’ (Gi = 90.36) (Table 1).

In vitro cultures of *V. vinifera* cv. Solaris after 30 days of cultivation were characterized by good viability in each of the tested media variants. Biomass increments were average. Low development of shoots and leaves was observed in each of the variants. The shoots took on a light green color (Figure 1). The highest Gi value was found for cultures grown in the ‘W4’ medium variant (Gi = 89.50) (Table 1).

### 2.2. Metabolic Profiling and Relative Quantification of Metabolites

The semi-targeted metabolomic profiling using UPLC–MS [39] allowed the identification of 45 compounds, including 6 amino acids, 1 organic acid, 3 phenolic acids, 13 flavan-3-ols, 3-flavonols, and 19 stilbenoids (Table 2). Twenty-two metabolites were identified by comparison with pure standards (level 1 metabolite identification) according to Metabolomics Standards Initiative [40]. Twenty-three metabolites were putatively annotated with level 2 identification and were identified according to elution order, UV spectra, and MS data from the literature (Table 2).

**Figure 2:**
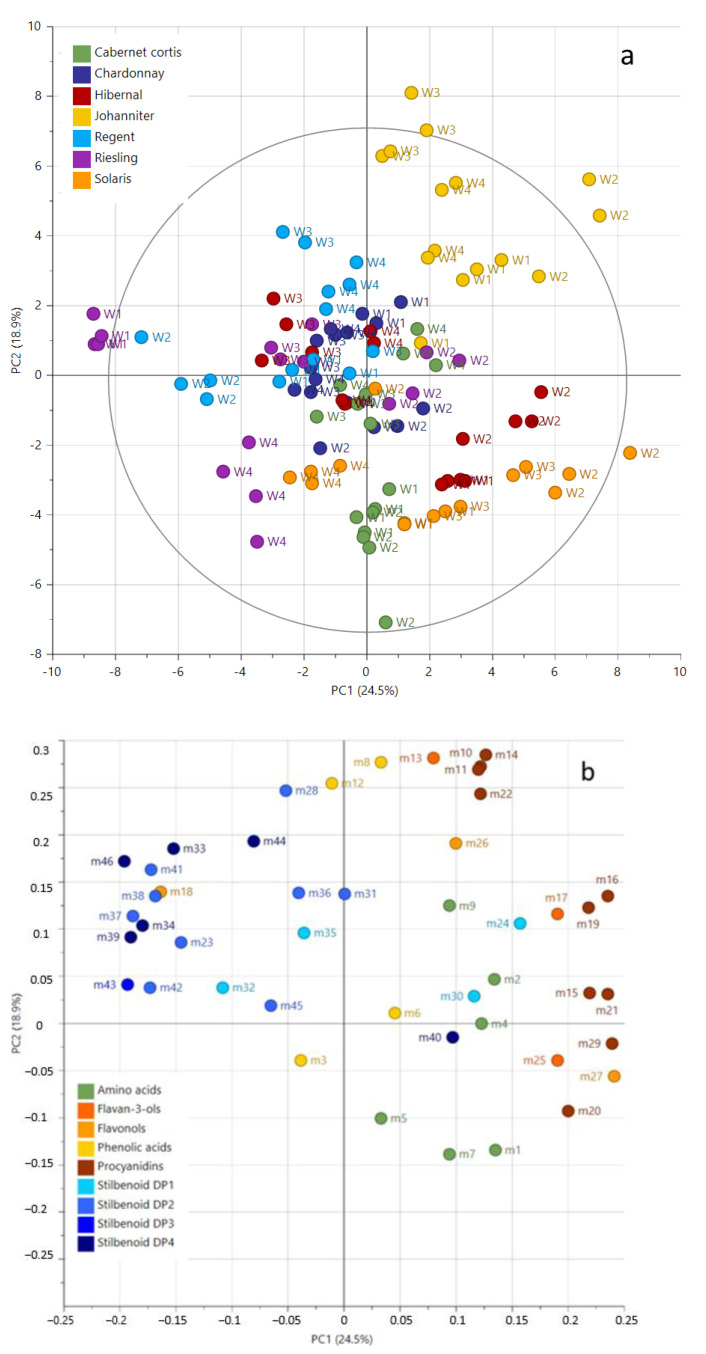
Unsupervised classification using principal component analysis (PCA) of the relative concentration of 45 metabolites from in vitro shoot cultures of *Vitis vinifera* cultivars under different culture conditions. Score plot (**a**) and loading plot (**b**). ‘W1’—MS, 0.9 mg/L BA and 0.1 mg/L IBA, ‘W2’—MS, 1.5 mg/L BA and 0.2 mg/L NAA, ‘W3’—SH, 0.9 mg/L BA and 0.1 mg/L IBA, ‘W4’—SH, 1.5 mg/L BA and 0.2 mg/L NAA. Numbers indicate the ID of the compounds as given in Table 2.

The relative quantification of the selected compounds was performed using selected ion monitoring (SIM) generated chromatograms. The corresponding peak areas were subjected to principal component analyses (PCA). PCA was performed to show the principal differences in the metabolite composition of *V. vinifera* in vitro shoot cultures from different cultivars under different culture conditions. The PCA score plot of the two first components explained 43.4% of the variation (Figure 2). The samples were clearly separated according to genotype and in second order to the culture conditions on the score plot (Figure 2a).

The phytochemical composition of Johanniter extracts was always separated independent of culture conditions. Other extracts were discriminated under specific culture conditions, for instance, Riesling extracts in ‘W4’ or Solaris extracts in ‘W2’ showed a strong influence on the phytohormonal and metabolic composition of the media. The basal medium, i.e., SH (‘W1’ and ‘W2’) or MS (in ‘W3’ and ‘W4’), also influenced the phytochemical composition of the extracts. The corresponding loading plot (Figure 2b) showed the variables responsible for the sample separation with a projection of stilbenoids DP1-3 as PC1 negative, but procyanidins, flavan-3-ols, and amino acids are PC1 positive. For example, extracts from Riesling in ‘W1’ and ‘W4’ and from Regent in ‘W4’ showed higher amounts of stilbenoids DP1-3 but poor amounts of procyanidins, flavan-3-ols, and amino acids. The opposite pattern was observed in extracts from Johanniter, Hibernal, and Solaris in ‘W2’.

#### Quantitative Analysis of Metabolites

The quantification of 45 compounds in the studied extracts with UPLC–MS analyses shows the result of how the growing conditions of specific grape vine cvs. affect their metabolic profile. The experimental outcomes are fully innovative. The specific data for each metabolite are given in the Supplementary materials (Table S1). Table 3 illustrates the total content of specific metabolite groups (amino acids, organic acids, phenolic acids, flavon-3-ols, flavonols, and stilbenoids DP1, DP2, DP3, and DP4) across all seven varieties cultivated in four variants of culture media (‘W1’–‘W4’). The tested extracts showed a high content of amino acids. The total amino acid content was the highest for the Cabernet Cortis cv. grown in ‘W2’ medium, 90.42 mg/g DW, and the lowest for the Riesling cv. in ‘W3’ medium, 12.39 mg/g DW. Therefore, bioproduction of amino acids is stimulated most in the growth conditions of ‘W1’ and ‘W2’ and the least in the ‘W3’ medium (Table 3). The amino acid whose content was the highest in all tested extracts was *L*-phenylalanine (from 11.50 mg/g DW to 88.37 mg/g DW for Cabernet Cortis cv. in medium ‘W2’ and Riesling in medium ‘W3’, respectively) (Table S1). Interestingly, the most effective biosynthesis of organic acids was influenced by ‘W4’ medium, and the content of these compounds ranged from 1.34 mg/g DW to 3.02 mg/g DW (for Johanniter and Solaris cvs., respectively) (Table 3). Relatively high contents of metabolites were also determined for flavan-3-ols and resveratrol dimers (DP2 stilbenoids). The content of flavan-3-ols ranged from 5.37 mg/g DW to 10.07 mg/g DW (for Riesling and Johanniter cvs. in medium ‘W2’, respectively). Resveretarol dimer (DP2) concentration was highest in the extract from the Hibernal cv. grown in ‘W1’ medium and the lowest in the Chardonnay cv. maintained in ‘W2’ medium (6.48 mg/g DW and 2.13 mg/g DW, respectively). The most potent media for the bioproduction of stilbenoids were ‘W2’ (3.14 mg/g DW and 2.53 mg/g DW for Rielsling and Joahanniter cvs., respectively) as well as ‘W4’ (2.67 mg/g DW for Hibernal cv.) (Table 3). The lowest concentrations were seen in varieties cultured in the ‘W3’ medium (from 22.96 mg/g DW to 51.84 mg/g DW for the Riesling and Regent cvs., respectively), while the highest content was found in the varieties grown in the ‘W2’ medium (from 55.98 mg/g DW to 100.81 mg/g DW, respectively, for Riesling and Cabernet Cortis cvs.) (Table 3).

### 2.3. Biological Activities

#### 2.3.1. Antioxidant Activity

To determine the primary antioxidant activity, the DPPH (1,1-diphenyl-2-picrylhydrazyl) assay was used. Ferrous ion (Fe^2+^) chelating assay was used to indicate the secondary antioxidant properties by measuring the capacity of extracts to chelate metal ions [48].

The results of the DPPH assay showed that all tested extracts exhibited antioxidant activity. Potency was dependent on the culture medium type: MS (‘W1’, ‘W2’) and SH (‘W3’, ‘W4’) (Table 1). Among all tested extracts, the best results were obtained for cv. Johanniter in ‘W1’ and ‘W2’ (MS) media with values of 33.57% and 31.15%, respectively. The cv. Riesling cultured in ‘W2’ medium was also effective, having an inhibition value of 28.89%. Notable results were also obtained for cv. Chardonnay cultured in ‘W2’ and ‘W4’ culture media with values of 23.61% and 21.77%, respectively. Regardless of the culture medium, the lowest efficiency was seen in cv. Solaris with values ranging from 2.07 to 5.46%. There was no significant impact of the culture media on the scavenging activity of cv. Regent (Table 2). According to the results, slight changes were also obtained for cv. Cabernet Cortis, while cv. Hibernal showed better efficiency when cultured in the ‘W3’ medium variant (11.49%) (Table 4).

The results of the ferrous ions’ (Fe^2+^) chelating ability assay showed a marked influence of the extracts on the iron–ferrozine formation complex. Among the tested extracts, the highest effects were obtained for cv. Cabernet Cortis with a chelating activity value of 50.93% cultured in the ‘W2’ medium (MS). The cv. Chardonnay cultured in the ‘W2’ medium also exhibited significant chelating activity with a value of 43.21%. A significant impact was also observed for cv. Regent (Table 1). The best results for cv. Johanniter and cv. Hibernal were obtained in the ‘W3’ medium (SH) with chelating activities of 32.66% and 19.06%, respectively, while cv. Riesling was more potent in the ‘W4’ medium (35.18%). Regardless of the culture medium, cv. Solaris showed nearly the same inhibitory activity ranging from 13.84 to 18.11% (Table 4). When comparing the obtained DPPH results to the reference sample, Trolox (125 µg/mL), with an activity reaching 73.73%, it becomes evident that the vine extracts exhibit lower oxidative activities (up to 33.57% for cv. Johanniter). Similarly, in terms of chelating properties, the results show a slight decrease (as opposed to 5 mM EDTA solution), a maximum 50.93% inhibition for Cabernet Cortis and 97.14% for the reference sample. Nevertheless, these activities are still sufficiently high for the tested samples to effectively diminish free radicals. The inhibition of oxidation plays a critical role in preventing the degradation of skin protein structures and the oxidation of lipids. These processes are responsible for maintaining the overall integrity of both the epidermis and dermis layers.

#### 2.3.2. Tyrosinase Inhibition Activity

Among tested extracts, the highest results of whitening potential were observed for cv. Hibernal cultured in ‘W4’ medium, having a tyrosinase inhibiting activity value of 17.50%. The cv. Johanniter and cv. Solaris in ‘W3’ medium also exhibited significant tyrosinase inhibition capacity with values of 16.29% and 16.23%, respectively. The most remarkable impact of the tested media was obtained for cv. Cabernet Cortis cultured in ‘W2’ medium (MS) with a value of 15.49% while the best results for cv. Riesling were obtained for shoots cultured in medium ‘W1’ (11.64%). Results clearly indicate that extracts obtained from grapevine in vitro cultures are active tyrosinase inhibitors (Table 5). All the tested extracts exhibit moderate activity in the inhibition of tyrosinase, leading to a decrease in skin discoloration. Tyrosine serves as a natural precursor to melanin and excessive production of can lead to hyperpigmentation. Tyrosinase acts as the pivotal enzyme responsible for converting tyrosine into the skin’s inherent pigment. Blocking this process could be an efficacious approach, not just for averting the emergence of “age spots” and skin discoloration, but conceivably for melanoma prevention. Notably, there was no correlation between the anti-tyrosinase inhibition and medium type. There was also no impact of grapevine metabotype on the ability for potential skin discoloration, however, Hibernal cv. exhibited the highest skin whitening potential. On the other hand, the results obtained for kojic acid (10 µg/mL) were comparable to tested extracts (11.05%). This underscores the considerable potential of the tested extracts as cosmetic ingredients with skin brightening effect.

## 3. Discussion

Plant secondary metabolites are important substances exerting a wide range of biological effects [49]. However, external factors have an extreme impact on the concentration of valuable chemical constituents in plants and influence its biological activity. For this reason, in vitro cultures could be used as an alternative source of valuable metabolites. In our study, we investigated the influence of several types of agar culture media of Murashige and Skoog (MS) and Schenk and Hildebrandt (SH) with supplementation of different plant growth regulators (PGRs) on the phytochemical profile and biological activities of biomass extracts. 

In order to compare the qualitative composition of in vitro plants, UPLC–MS analyses were performed. We cultured seven grape varieties across four media and analyzed amino acids, organic acids, phenolic compounds, flavonoids, and stilbenoids. To the best of our knowledge, it is the first wide and complex study of its kind performed on shoot cultures of different *V. vinifera* cvs. The quantitative results showed the influence of culture conditions on shoot cultivation and type of grapevine cvs. on the production of 45 detected compounds (Table 3 and Supplementary Materials). Amino acid content stands out across all groups, with Cabernet Cortis cv. exhibiting the highest content (90.42 mg/g DW) in ‘W2’ medium and Riesling the lowest (12.39 mg/g DW) in ‘W3’ medium. ‘W2’ and ‘W1’ media stimulate the highest amino acid bioproduction, while ‘W3’ medium results in the least. *L*-Phenylalanine prevails as the most abundant amino acid in all extracts. Organic acid synthesis is most effective under ‘W4’ medium conditions, varying from 1.34 mg/g DW to 3.02 mg/g DW across different varieties. Flavan-3-ols and DP2 stilbenoids (resveratrol dimers) also show relatively high content. Flavan-3-ols range from 5.37 mg/g DW to 10.07 mg/g DW, with Hibernal cultivar in ‘W1’ medium exhibiting the highest resveratrol dimer concentrations. ‘W2’ and ‘W4’ media stand out for stilbenoid bioproduction, while ‘W3’ medium demonstrates the lowest overall metabolite concentration. The lowest metabolite concentration overall is observed in ‘W3’ medium (22.96 mg/g DW to 51.84 mg/g DW), while the highest content appears in varieties cultivated in ‘W2’ medium (55.98 mg/g DW to 100.81 mg/g DW) (Table 3 and Supplementary Materials). Quantitative analysis reveals how grape cvs. metabolic profiles are influenced by growth conditions, emphasizing amino acid, organic acid, phenolic compound, flavonoid, and stilbenoid contents. While specific medium–metabolite correlations are limited, the research underscores the impact of certain media on particular metabolite groups and highlights the significant role of amino acids across all tested compounds. The findings contribute to a deeper understanding of the intricate relationship between grape growth conditions and metabolite production.

PCA analysis showed grapevine cultivar impacts the final metabolite composition of the extracts more than the medium.

The highest Gi coefficient for in vitro cultures of *V. vinifera* was found in cv. Chardonnay cultured in ‘W2’ medium (Gi = 95.89). Satisfactory results were obtained for cv. Cabernet Cortis (Gi = 92.24), cv. Johanniter (Gi = 91.83), and cv. Hibernal (Gi = 90.94) also cultured in ‘W2’ medium. When the Gi growth factor was compared for all tested cultivars, the best condition for the development of in vitro cultures of *V. vinifera* cvs. was found to be media variant ‘W2’ (MS + 1.5 mg/mL BA and 0.2 mg/mL NAA). Further, the addition of cytokinin BA and auxin NAA promotes the growth and development of *V. vinifera* in vitro cultures.

To determine primary antioxidant activity, we performed the DPPH (1,1-diphenyl-2-picrylhydrazyl) assay, while secondary antioxidant properties were measured using the ferrous ion chelating assay. Our findings showed that all tested extracts exhibit radical scavenging activity dependent on the applied media. In the DPPH assay, cv. Johanniter in ‘W1’ (MS) media was found to be the most effective (inhibition value 33.57%). The results of the ferrous ion chelating assay showed that the extracts had good chelating activity. The cv. Cabernet Cortis cultured in ‘W2’ medium (MS) showed the strongest chelating ability (50.93%). The evaluation of *V. vinifera* in vitro cultures’ antioxidant activity revealed significant protective abilities of its cultivars against free radicals and reactive oxygen species. Results also suggest that the antioxidant activity of *V. vinifera* in vitro extracts could be attributed to the identified rich phytochemical profile.

We performed tyrosinase inhibition assay to investigate the skin-whitening potential of the tested *V. vinifera* in vitro cultivars. The best results were obtained for cv. Hibernal cultured in ‘W4’ medium (SH) (17.50%). *V. vinifera* in vitro cultivars could be potential raw materials for skin-whitening formulations. Comparing the results obtained in our previous studies, the extracts tested were slightly less active than the samples obtained from conventional crops (from 30.4 for Sauvignon to 62.5% for Rielsling) [18]. However, the previous studies contained cane extracts as woody biomass (unlike in vitro samples). Cane extracts are widely recognized for the abundance and diversity of compounds within the stilbenoid group. These serve as the principal metabolites responsible for inhibiting the tyrosinase enzyme. Hence, a comparison based on these results may not be meaningful.

The activity of grapevine in vitro cultures is a novel and unexplored topic. Further research may allow for a reliable assessment of these raw materials as multifunctional active ingredients in cosmetics. The great benefit of using this breeding method is independence from environmental conditions, obtaining reproducible results due to the defined and constant composition of the raw material, and the possibility of eliminating many pesticides and plant protection agents that are not desirable in raw materials dedicated for cosmetic purposes.

## 4. Materials and Methods

### 4.1. Chemicals and Reagents

Reagents needed for cultivation of in vitro cultures, e.g., MS and SH basal media, agar, PGRs, and sucrose were acquired from Duchefa Farma B.V. (Haarlem, The Netherlands).

Ultrapure water was obtained using Millipore Milli-Q system (Merck Millipore, Molsheim, France). Acetonitrile and methanol were purchased from Thermo Fisher Scientific (Illkirch-Graffenstaden, France). Standard compounds *L*-leucine, *L*-isoleucine, *L*-phenylalanine, *L*-tyrosine, *L*-tryptophan, *L*-proline, citric acid, gallic acid, coutaric acid, *trans*-resveratrol, *cis*-resveratrol, *E*-piceid, catechin, epicatechin, catechin gallate, tyrosinase, and *L*-DOPA were purchased from Sigma-Aldrich (St. Louis, MO, USA). Rhamnetin, *E*-ε-viniferin, quercetin 3-*O*-glucoside, quercetin 3-*O*-glucuronide, quercetin-3-O-galactoside, procyanidin B1, procyanidin B2, procyanidin B3, and procyanidin C1 were obtained from ExtraSynthèse (Genay, France); caftaric acid was delivered by Carbosynth (Compton, Berkshire, UK), while *E*-ε-viniferin, *Z*/*E*-vitisin B, ampelopsin A, and hopeaphenol were obtained by previous extraction from grape stems [50].

### 4.2. In Vitro Cultures Initiation

*Vitis vinifera* L. different cvs., five white varieties: cv. Chardonnay, cv. Hibernal, cv. Riesling, cv. Johanniter, and cv. Solaris and two red ones: cv. Cabernet Cortis and cv. Regent, were used for the experiment. For culture initiation, the young stems with buds were collected in June 2021 from the vineyard “Srebrna Góra” (Al. Konarowa 1, Kraków, 30-248; https://.winnicasrebrnagora.pl, accessed on 30 August 2023).

The fragments of vine stems with buds 1–3 cm long were sterilized for 5 min with 0.2% mercuric chloride (HgCl_2_), washed with distilled water three times, and placed on Murashige and Skoog (MS) medium supplemented with 10 mg/L of vitamin B1 and 0.9 mg/L 6-benzyladenine (BA) and 0.3 mg/L indole-3-butyric acid (IBA).

In vitro cultures were maintained under artificial white LED light conditions (88 ± 8 mol × m^−2^ × s^−1^; Philips-Flora TL-D 35W/33 fluorescent lamps, Philips, France) without photoperiod. The temperature was kept at 23 ± 2 °C.

### 4.3. Experimental In Vitro Cultures

Agar (7.2% *v*/*v*) variants of ‘W1’ and ‘W2’ of the MS medium and ‘W3’ and ‘W4’ of the SH medium were tested for in vitro *V. vinifera* microshoot cultures of seven cultivars: cv. Johanniter, cv. Chardonnay, cv. Riesling, cv. Cabernet Cortis, cv. Hibernal, cv. Regent, and cv. Solaris. The weight of the inoculum was between 0.5 and 1.0 g fresh weight. The cultures were carried out in 3 repetitions (3 series) of 3 jars per medium variant. Two types of culture media were tested—Murashige and Skoog (MS) [51] and Shenk-Hildebrandt (SH) [52]. The culture media differed in the composition of plant growth and development regulators (PGRs) and were marked as ‘W1’ and ‘W2’ for MS medium and ‘W3’ and ‘W4’ for SH medium. Variants ‘W1’ and ‘W2’ were based on the MS medium (Murashige and Skoog). ‘W1’ is the variant of the MS medium supplemented with PGRs 0.9 mg/L BA and 0.3 mg/L IBA, and ‘W2’ is the variant of the MS medium supplemented with PGRs 1.5 mg/L BA and 0.2 mg/L NAA. Variants ‘W3’ and ‘W4’ were based on the SH medium (Schenk and Hildebrandt). Variant ‘W3’ contained the addition of PGRs 0.9 mg/L BA and 0.3 mg/L IBA, and ‘W4’ contained the addition of PGRs 1.5 mg/L BA and 0.2 mg/L NAA.

The biomass was collected, rinsed in distilled water, frozen, and lyophilized (LABCONCO lyophilizer, Kansas City, MO, USA). Based on dry weight, biomass increments were calculated using the Gi coefficient according to the formula:

$$\;\;G_{i}=\frac{D_{w_{n}}-D_{w_{0}}}{D_{w_{n}}}\times 100$$

where *Gi*—growth index; *Dw_0_*—dry weight of inoculum; and *Dw_n_*—dry weight after *n* time.

In vitro cultures of *V. vinifera* were carried out under conditions of constant artificial illumination with white LED light with an intensity of 4 W/m^2^. The temperature was maintained at 23 ± 2 °C.

### 4.4. Metabolite Profiling

#### 4.4.1. Extraction

For UPLC–MS analyses, the extraction of dried biomass was performed using ultrasound assisted extraction. For this, 20 mg of the dry plant material was placed in Eppendorf with 1 mL of 70% ethanol (*v*/*v*). The samples were prepared in quintuplicates. The extraction was performed for 30 min at 4 °C in darkness. Then, samples were centrifuged at 18,000 rpm for 10 min at 4 °C. Finally, 50 µL on the supernatant was transferred to vials prior UPLC/MS analyses.

#### 4.4.2. UPLC–MS Analyses

The UPLC–MS system used an ACQUITY™ Ultra Performance Liquid Chromatography system coupled with a photo diode array detector (PDA) and a Xevo TQD mass spectrometer (Waters, Milford, MA, USA) equipped with an electrospray ionization (ESI) source controlled by Masslynx 4.1 software (Waters, Milford, MA, USA). A Waters Acquity HSS T3 C18 column (150 × 2.1 mm, 1.8 μm) ensured analyte separation with a flow rate of 0.4 mL min^−1^ at 55 °C. The injection volume was 5 μL. The mobile phase was made of 0.1% formic acid in water (solvent A) and 0.1% formic acid in acetonitrile (solvent B). The chromatographic separation was performed using an 18-min linear gradient from 5–50% solvent B followed by washing and column reconditioning for 8 min. MS detection was performed in both positive and negative modes. The capillary voltage was 3000 V and sample cone voltages were 30 and 60 V. The cone and desolvation gas flow rates were 60 and 800 Lh^−1^, respectively. Metabolite profiling was performed according to retention times, MS, and UV spectra with comparison to standards. For relative quantification, UPLC–MS analyses were performed in selected ion monitoring (SIM) mode, and the resulting SIM chromatograms were integrated using the ApexTrack algorithm with a mass window of 0.1 Da and relative retention time window of 0.2 min followed by Savitzky–Golay smoothing (iteration = 1, width = 1) using Targetlynx software (v.4.2; Waters, Milford, MA 01757, USA). The resulting peak integrations and retention times were visually examined. The robustness of the measurements and analytical variability were evaluated through a series of quality control (QC) samples prepared by pooling all samples and injected before, during, and after the batch. Samples were injected at random independent of genotype or culture conditions. Detected compounds are expressed in mg/g DW (dry weight).

#### 4.4.3. Statistical Analysis

Multivariate analysis (MVA) was performed using SIMCA P+ version 17.0 (Umetrics AB, Umeå, Sweden). Variables were mean-centered and unit-variance scaled prior to MVA. Principal component analyses (PCA) was used as unsupervised MVA to visualize the influence of genotype, basal medium, and phytohormonal composition on the phytochemical composition.

### 4.5. Evaluation of Biological Activity

#### 4.5.1. Extraction

For biological investigations, the samples were extracted in 70% ethanol in ultrasonic bath (Hettig, Solingen, Germany) for 30 min at a sample/ethanol ratio 1 g/10 mL of solvent. Then the samples were lyophilized (Labconco, Kansas, MO, USA). The extraction rates for individual cultivars varied from 12% to 15% by mass. The obtained dry extracts were dissolved in 0.1% ethanol (*v*/*v*) using the ratio of 25 mg of dry extract/100 g of solvent. Samples were dissolved in ethanol, then diluted in water. Extracts were subjected to antioxidant tests and tyrosinase assays.

#### 4.5.2. Antioxidant Activity

##### Free Radical Scavenging Activity

The free radical scavenging activity was estimated using the DPPH assay [48]. Briefly, an aliquot (0.5 mL) of each extract was mixed with 3 mL of 0.1 mM of freshly prepared methanolic 5 mM DPPH solution and incubated for 20 min. The control samples comprised methanol and DPPH solution without extracts. The absorbance was measured at a wavelength of 517 nm using a spectrophotometer (Nanocolor^®^ UV/VIS, Macherey Nagel). The radical scavenging activity was averaged from three independent experiments and calculated based on the following formula [53]:

% inhibition = [(A_c_ − A_s_)/(A_c_)] × 100

where A_c_—absorbance of control sample and A_s_—absorbance of tested sample.

##### Ferrous Ion (Fe^2+^) Chelating Activity

The Fe^2+^ chelating activity of *V. vinifera* extracts was evaluated by measuring the formation of Fe^2+^–ferrozine complex according to the method previously described [48]. Briefly, 1 mL of each extract was mixed with 0.5 mL of methanol and 0.05 mL of FeCl_2_ (2 mM). The complex formation was initiated by adding 0.1 mL of ferrozine (5 mM). The absorbance of samples was measured at 562 nm using Nanocolor UV/VIS spectrophotometer (Macherey Nagel). The obtained results were averaged from three independent results and expressed as an inhibition percentage of the Fe^2+^–ferrozine complex formation (%):

% chelating = [(A_c_ − A_s_)/(A_c_)] × 100

where A_c_—absorbance of control sample and A_s_—absorbance of tested sample.

#### 4.5.3. Tyrosinase Inhibition Activity

The anti-tyrosinase activity was assessed using the previously described method [18]. Briefly, 50 μL of extracts, 80 μL of phosphate buffer (67 mM, pH 6.8), and 40 μL of tyrosinase were pipetted into a 96-well plate and incubated for 5 min at 25 °C. Kojic acid (25, 50, 100 μg/mL) was used as a positive control. Then, 40 μL of L-DOPA was added to the wells. The absorbance was measured at a wavelength of 475 nm using a microplate reader (Tecan). The results were averaged from two repeated experiments. The tyrosinase inhibitory activity was expressed as and calculated as follows:

% inhibition = [(slopeA_c_ − slopeA_s_)/(slopeA_c_)] × 100

## 5. Conclusions

In the presented study, we performed initiation and optimization of seven cultivars of *V. vinifera*: cv. Johanniter, cv. Chardonnay, cv. Riesling, cv. Cabernet Cortis, cv. Hibernal, cv. Regent, and cv. Solaris. Optimization of agar shoot cultures included testing different culture media, MS (Murashige and Skoog) and SH (Schenk and Hildebrandt), and different compositions of PGRs. Four media variants were tested: ‘W1’, ‘W2’, ‘W3’, and ‘W4’.

Based on the comparison of the Gi growth factor, it was found that the best conditions for the development of in vitro cultures of *V. vinifera* cvs. was media variant ‘W2’ (MS + 1.5 mg/mL BA and 0.2 mg/mL NAA). The smallest increases in biomass were found in the ‘W1’ medium (MS + 0.9 mg/mL BA and 0.1 mg/mL). 

The antioxidant potential was determined using the DPPH test, ferrous ion (Fe^2+^) chelation, and tyrosinase inhibition of extracts from in vitro cultures of *V. vinifera*. All the extracts tested exhibited relative strong antioxidative activity; however, there is no correlation between their ability to reduce free radicals and their chelating activity. The highest % inhibition in the DPPH assay was obtained for cv. Johanniter cultured in ‘W1’ and ‘W2’ (MS) media with values of 33.57% and 31.15%, respectively. However, the highest % ferrous ion chelating capacity was revealed to be cv. Cabernet Cortis with a chelating activity value of 50.93% when cultured in ‘W2’ medium (MS). The strongest tyrosinase inhibition potential was obtained for cv. Hibernal cultured in ‘W4’ medium (SH) with the inhibiting activity value of 17.50%. The relative high antioxidant activity and the ability to neutralize metal ions make the extracts tested interesting ingredients in skin care formulations. Phenolic acids and flavan 3-ols have the highest tyrosinase inhibitory effect. We conclude the most potent *V. vinifera* cultivars were cv. Johanniter, cv. Cabernet Cortis, and cv. Hibernal cultured in different types of media. Our findings could provide valuable information for further investigation using the above-mentioned cultivars. These studies are the first to elucidate the benefits of grapevine cvs. shoot culture extracts as potentially effective, multifunctional cosmetic ingredients, which can protect skin from oxidative stress as well as hyperpigmentation.

## Figures and Tables

**Table 1:** Gi values of shoot cultures biomass of various *V. vinifera* cvs. using variations of culture media (‘W1’—MS, 0.9 mg/L BA and 0.1 mg/L IBA, ‘W2’—MS, 1.5 mg/L BA and 0.2 mg/L NAA, ‘W3’—SH, 0.9 mg/L BA and 0.1 mg/L IBA, ‘W4’—SH, 1.5 mg/L BA and 0.2 mg/L NAA).

*V. vinifera* cvs.	Medium Variant			
	W1	W2	W3	W4
**Johanniter**	89.95 ± 1.37	91.83 ± 0.03	88.03 ± 4.40	88.44 ± 1.57
**Chardonnay**	87.77 ± 6.95	95.89 ± 4.79	91.85 ± 4.58	92.73 ± 1.98
**Riesling**	84.85 ± 5.45	94.99 ± 6.78	95.26 ± 8.55	94.95 ± 3.89
**Cabernet Cortis**	86.05 ± 8.55	92.24 ± 5.12	84.44 ± 6.45	91.10 ± 9.43
**Regent**	85.67 ± 2.47	73.78 ± 4.78	89.60 ± 3.42	90.36 ± 7.14
**Hibernal**	81.60 ± 7.58	90.94 ± 3.80	81.86 ± 4.21	89.75 ± 7.61
**Solaris**	82.98 ± 3.36	86.71 ± 2.74	83.17 ± 3.33	89.50 ± 5.49

**Table 2:** List of identified metabolites from the studied *V. vinifera* in vitro culture extracts.

ID Metabolite	Metabolic Class	RT	Name	Authentication
m1	Amino acids	1.07	*L*-Proline	Standard
m2		1.37	*L*-Tyrosine	Standard
m4		1.54	*L*-Isoleucine	Standard
m5		1.67	*L*-Leucine	Standard
m7		2.45	*L*-Phenylalanine	Standard
m9		3.56	*L*-Tryptophan	Standard
m3	Organic acids	1.38	Citric acid	Standard
m6	Phenolic acids	1.85	Gallic acid	Standard
m8		3.36	Caftaric acid	Standard
m12		4.29	Coutaric acid	Standard
m25	Flavan-3-ols	7.06	Catechin gallate	[41]
m13		4.38	Catechin	Standard
m17		5.44	Epicatechin	Standard
m10		3.89	Procyanidin B1	Standard
m11		4.21	Procyanidin B3	Standard
m15		4.78	Procyanidin B4	Standard
m16		4.91	Procyanidin B2	Standard
m22		6.01	Procyanidin dimer 5	[42]
m20		5.82	Galloyl Procyanidin B _a_	[43]
m21		5.97	Galloyl Procyanidin B _b_	[43]
m29		8.31	Galloyl Procyanidin B _c_	[43]
m14		4.44	Procyanidin trimer	[42]
m19		5.70	Procyanidin C1	Standard
m18	Flavonols	5.65	Quercetin-hexoside	[21]
m26		7.33	Quercetin glucuronide	[21]
m27		7.44	Quercetin-3-*O*-glucoside	Standard
m35	Stilbenoids DP1 *	10.79	*Z*-Resveratrol	Standard
m32		9.39	*E*-Resveratrol	Standard
m24		7.01	Piceid	Standard
m23	Stilbenoids DP2 *	6.16	Restrytisol 1	[44]
m28		7.54	Ampelopsin A	Standard
m31		8.72	Resveratrol dimer glycosylate	[45]
m37		11.62	*Z-*ε-Viniferin	[44]
m38		11.86	*E*-ε-Viniferin	Standard
m41		12.82	α-Viniferin	[46]
m42		13.30	ω-Viniferin	[47]
m45		14.20	δ-Viniferin	[44]
m36	Stilbenoids DP3 *	11.05	Resveratrol trimer 1	[45]
m43		13.99	Resveratrol trimer 2	[45]
m33	Stilbenoids DP4 *	10.39	Hopeaphenol	Standard
m34		10.63	Isohopeaphenol	[45]
m39		12.01	Resveratrol tetramer 3	[45]
m40		12.60	Resveratrol tetramer 4	[45]
m44		14.14	Vitisin B	Standard
m46		14.52	Resveratrol tetramer 6	[45]

* DP1—monomeric; DP2—dimeric; DP3—trimeric; DP4—tetrameric. _a, b, c_—three different structures of Proanthocyanidin B, defined by an exact framework.

**Table 3:** Total content of identified metabolites in the extracts of shoot cultures of different *V. vinifera* cvs. Table corresponds with the Supplementary Materials. Values are expressed as mg/g DW (±SD, n = 4).

*V. vinifera* cvs.	Medium Variant *	Amino Acids	Organic Acids	Phenolic Acids	Flavan-3-ols	Flavonols	Stilbenoids DP1	Stilbenoids DP2	Stilbenoids DP3	Stilbenoids DP4	Total Metabolite Content
**Johanniter**	W1	23.14 ± 0.60	0.58 ± 0.1	1.44 ± 0.08	7.04 ± 0.09	0.31 ± 0.02	2.17 ± 0.13	3.57 ± 0.06	0.49 ± 0.02	0.38 ± 0.00	39.1 ± 0.12
	W2	53.69 ± 0.45	0.55 ± 0.07	1.01 ± 0.02	10.07 ± 0.12	0.35 ± 0.01	2.53 ± 0.09	5.83 ± 0.04	0.54 ± 0.06	0.37 ± 0.01	74.93 ± 0.1
	W3	16.09 ± 0.28	1.12 ± 0.13	1.04 ± 0.04	7.90 ± 0.07	0.28 ± 0.01	1.40 ± 0.07	4.53 ± 0.04	0.64 ± 0.04	1.38 ± 0.02	34.39 ± 0.07
	W4	21.81 ± 0.51	1.34 ± 0.22	0.91 ± 0.05	7.53 ± 0.07	0.23 ± 0.01	2.42 ± 0.12	6.10 ± 0.09	0.76 ± 0.04	0.67 ± 0.02	41.76 ± 0.11
**Chardonnay**	W1	45.91 ± 0.84	0.76 ± 0.07	0.6 ± 0.00	5.43 ± 0.06	0.14 ± 0.01	1.57 ± 0.13	6.34 ± 0.05	0.62 ± 0.02	0.52 ± 0.01	61.89 ± 0.14
	W2	29.04 ± 0.19	0.67 ± 0.08	2.05 ± 0.04	3.69 ± 0.05	0.16 ± 0.01	1.35 ± 0.08	2.13 ± 0.05	0.33 ± 0.03	0.08 ± 0.00	39.49 ± 0.05
	W3	21.51 ± 0.45	1.68 ± 0.1	0.64 ± 0.03	4.20 ± 0.05	0.12 ± 0.01	0.87 ± 0.04	5.08 ± 0.05	0.69 ± 0.03	0.50 ± 0.01	35.29 ± 0.08
	W4	28.59 ± 0.69	1.81 ± 0.34	0.64 ± 0.04	3.79 ± 0.04	0.17 ± 0.01	0.61 ± 0.03	3.54 ± 0.05	0.50 ± 0.02	0.49 ± 0.01	40.14 ± 0.11
**Riesling**	W1	25.41 ± 0.46	0.41 ± 0.07	0.38 ± 0.01	1.98 ± 0.02	0.20 ± 0.01	1.21 ± 0.06	15.06 ± 0.08	1.06 ± 0.07	1.68 ± 0.04	47.4 ± 0.09
	W2	40.73 ± 0.92	0.95 ± 0.08	0.76 ± 0.01	5.34 ± 0.05	0.18 ± 0.01	3.14 ± 0.09	4.00 ± 0.08	0.56 ± 0.04	0.31 ± 0.01	55.98 ± 0.15
	W3	12.39 ± 0.18	1.03 ± 0.11	0.73 ± 0.04	3.04 ± 0.03	0.28 ± 0.02	1.20 ± 0.04	3.02 ± 0.05	0.32 ± 0.03	0.95 ± 0.02	22.96 ± 0.05
	W4	18.13 ± 0.48	2.07 ± 0.29	0.34 ± 0.01	0.70 ± 0.01	0.19 ± 0.01	0.52 ± 0.01	2.69 ± 0.04	0.45 ± 0.04	0.56 ± 0.01	25.66 ± 0.08
**Cabernet Cortis**	W1	63.06 ± 1.57	0.7 ± 0.04	0.14 ± 0.00	3.60 ± 0.04	0.08 ± 0.01	0.69 ± 0.03	5.59 ± 0.04	0.63 ± 0.03	0.24 ± 0.01	74.73 ± 0.22
	W2	90.42 ± 2.27	0.85 ± 0.08	0.15 ± 0.00	3.59 ± 0.04	0.05 ± 0.00	0.80 ± 0.08	4.33 ± 0.03	0.47 ± 0.05	0.15 ± 0.01	100.81 ± 0.31
	W3	23.14 ± 0.44	1.75 ± 0.25	0.3 ± 0.00	5.77 ± 0.04	0.10 ± 0.01	0.36 ± 0.02	4.58 ± 0.08	0.46 ± 0.02	0.68 ± 0.01	37.14 ± 0.08
	W4	28.99 ± 0.78	1.96 ± 0.26	0.16 ± 0.00	8.33 ± 0.10	0.11 ± 0.01	0.93 ± 0.05	7.11 ± 0.11	1.09 ± 0.12	0.65 ± 0.02	49.34 ± 0.15
**Hibernal**	W1	33.96 ± 0.55	1.13 ± 0.17	0.24 ± 0.01	6.40 ± 0.04	0.18 ± 0.01	1.14 ± 0.05	6.48 ± 0.07	0.78 ± 0.04	0.24 ± 0.01	50.54 ± 0.1
	W2	51.62 ± 0.74	0.87 ± 0.04	0.25 ± 0.01	8.27 ± 0.08	0.17 ± 0.01	2.63 ± 0.16	5.42 ± 0.04	0.62 ± 0.04	0.26 ± 0.01	70.13 ± 0.14
	W3	23.66 ± 0.37	1.93 ± 0.36	0.77 ± 0.03	5.44 ± 0.05	0.16 ± 0.01	1.19 ± 0.08	9.21 ± 0.06	1.44 ± 0.08	0.77 ± 0.01	44.58 ± 0.09
	W4	29.58 ± 0.90	2.31 ± 0.35	0.35 ± 0.03	5.94 ± 0.08	0.16 ± 0.01	2.67 ± 0.23	6.53 ± 0.13	1.07 ± 0.11	0.43 ± 0.01	49.03 ± 0.19
**Regent**	W1	53.02 ± 1.55	0.76 ± 0.11	0.45 ± 0.00	5.33 ± 0.06	0.14 ± 0.01	1.57 ± 0.11	6.46 ± 0.06	0.83 ± 0.06	1.04 ± 0.02	69.6 ± 0.23
	W2	45.04 ± 0.65	1.79 ± 0.28	0.19 ± 0.01	3.52 ± 0.02	0.07 ± 0.00	1.54 ± 0.07	15.80 ± 0.08	1.73 ± 0.15	1.19 ± 0.02	70.87 ± 0.12
	W3	32.86 ± 0.06	1.6 ± 0.35	0.69 ± 0.06	7.19 ± 0.09	0.23 ± 0.01	1.20 ± 0.11	5.73 ± 0.12	0.88 ± 0.06	1.46 ± 0.02	51.84 ± 0.07
	W4	39.81 ± 0.57	1.73 ± 0.33	0.2 ± 0.01	7.45 ± 0.07	0.11 ± 0.00	1.82 ± 0.12	9.97 ± 0.09	1.48 ± 0.05	1.13 ± 0.01	63.71 ± 0.12
**Solaris**	W1	47.32 ± 1.50	1.03 ± 0.19	0.12 ± 0.01	4.99 ± 0.05	0.10 ± 0.00	1.44 ± 0.06	3.54 ± 0.06	0.45 ± 0.03	0.25 ± 0.00	59.25 ± 0.21
	W2	83.81 ± 1.48	1.41 ± 0.33	0.36 ± 0.02	7.30 ± 0.07	0.25 ± 0.01	2.43 ± 0.16	3.75 ± 0.09	0.34 ± 0.04	0.11 ± 0.00	99.75 ± 0.23
	W3	24.97 ± 0.08	1.89 ± 0.3	0.11 ± 0.00	6.27 ± 0.07	0.17 ± 0.01	1.47 ± 0.09	3.30 ± 0.03	0.55 ± 0.02	0.38 ± 0.01	39.11 ± 0.04
	W4	29.26 ± 0.89	3.02 ± 0.43	0.17 ± 0.01	4.12 ± 0.05	0.14 ± 0.01	0.65 ± 0.05	4.12 ± 0.04	0.59 ± 0.06	0.56 ± 0.02	42.64 ± 0.14

* ‘W1’—MS, 0.9 mg/L 6-benzylaminopurine (BA) and 0.1 mg/L indole-3-butyric acid (IBA), ‘W2’—MS, 1.5 mg/L BA and 0.2 mg/L 1-naphthaleneacetic acid (NAA), ‘W3’—SH, 0.9 mg/L BA and 0.1 mg/L IBA and ‘W4’—SH, 1.5 mg/L BA and 0.2 mg/L NAA.

**Table 4:** The free radical scavenging activity and ferrous ion (Fe^2+^) chelating ability of extracts obtained from shoot cultures of different *V. vinifera* cvs. Values are expressed as inhibition percentage (%). * ‘W1’–MS, 0.9 mg/L BA and 0.1 mg/L IBA, ‘W2’—MS, 1.5 mg/L BA and 0.2 mg/L NAA, ‘W3’—SH, 0.9 mg/L BA and 0.1 mg/L IBA, ‘W4’—SH, 1.5 mg/L BA and 0.2 mg/L NAA.

*V. vinifera* cvs.	Medium Variant *	DPPH Assay (Inhibition %)	Fe^2+^ Chelating Activity Assay (Inhibition %)
**Johanniter**	W1	33.57 ± 4.16	21.94 ± 3.45
	W2	31.15 ± 2.82	10.00 ± 0.20
	W3	19.99 ± 1.27	26.19 ± 1.44
	W4	14.16 ± 1.27	21.37 ± 4.74
**Chardonnay**	W1	15.96 ± 3.16	20.47 ± 4.14
	W2	23.61 ± 2.32	33.26 ± 1.86
	W3	6.33 ± 1.31	27.34 ± 3.25
	W4	21.72 ± 5.68	13.79 ± 1.57
**Riesling**	W1	2.64 ± 1.50	7.36 ± 0.52
	W2	28.89 ± 0.82	10.29 ± 3.07
	W3	17.63 ± 1.71	17.53 ± 3.06
	W4	6.48 ± 1.32	35.18 ± 0.15
**Cabernet Cortis**	W1	6.93 ± 1.50	27.48 ± 3.23
	W2	8.47 ± 1.42	50.93 ± 2.69
	W3	8.66 ± 1.03	32.75 ± 2.56
	W4	9.42 ± 0.79	28.11 ± 2.99
**Hibernal**	W1	8.44 ± 1.50	11.07 ± 0.01
	W2	1.02 ± 0.59	8.21 ± 3.77
	W3	11.49 ± 2.20	14.20 ± 3.32
	W4	6.70 ± 1.44	14.79 ± 5.20
**Regent**	W1	1.23 ± 0.5	1.23 ± 0.52
	W2	9.40 ± 0.47	8,31 ± 0,42
	W3	9.34 ± 0.40	15.39 ± 0.71
	W4	9.49 ± 0.74	2.91 ± 0.52
**Solaris**	W1	4.63 ± 1.50	18.11 ± 1.77
	W2	5.46 ± 1.50	17.09 ± 1.70
	W3	4.07 ± 0.41	14.83 ± 2.75
	W4	2.07 ± 0.19	13.84 ± 0.99
Reference sample *		73.73 ± 0.55	97.14 ± 0.15

* reference sample: DPPH assay: Trolox 125 µg/mL; Chelating ability: EDTA 125 µg/mL.

**Table 5:** The tyrosinase inhibition activity of extracts obtained from shoot cultures of different *V. vinifera* cvs. Values are expressed as inhibition percentage (%). * ‘W1’—MS, 0.9 mg/L BA and 0.1 mg/L IBA, ‘W2’—MS, 1.5 mg/L BA and 0.2 mg/L NAA, ‘W3’—SH, 0.9 mg/L BA and 0.1 mg/L IBA, ‘W4’—SH, 1.5 mg/L BA and 0.2 mg/L NAA.

*V. vinifera* cvs.	Medium Variant *	Tyrosinase Inhibition Activity (Inhibition %)
**Johanniter**	W1	8.90 ± 0.36
	W2	5.89 ± 0.24
	W3	16.29 ± 0.65
	W4	5.38 ± 0.22
**Chardonnay**	W1	12.27 ± 0.49
	W2	4.54 ± 0.18
	W3	11.64 ± 0.47
	W4	9.91 ± 0.40
**Riesling**	W1	11.64 ± 0.47
	W2	8.78 ± 0.35
	W3	9.87 ± 0.39
	W4	4.80 ± 0.19
**Cabernet Cortis**	W1	1.98 ± 0.08
	W2	15.49 ± 0.62
	W3	4.71 ± 0.19
	W4	9.86 ± 0.39
**Hibernal**	W1	10.39 ± 0.42
	W2	9.78 ± 0.39
	W3	8.78 ± 0.35
	W4	17.50 ± 0.70
**Regent**	W1	10.04 ± 0.40
	W2	10.61 ± 0.42
	W3	9.61 ± 0.38
	W4	7.45 ± 0.30
**Solaris**	W1	6.25 ± 0.25
	W2	7.43 ± 0.30
	W3	16.23 ± 0.65
	W4	7.32 ± 0.29
Reference sample *		11.05 ± 0.44

* reference sample: Kojic acid 100 µg/mL.

## Data Availability

Not applicable.

## Supplementary Materials

The following supporting information can be downloaded at: https://www.mdpi.com/article/10.3390/molecules28196868/s1. Table S1. The quantitative analysis results for the metabolites from shoot cultures of different *V. vinifera* cvs. Values are expressed as mg/g DW (±SD, n = 4).
