# Supplementary material for: Shoot Cultures of Vitis vinifera (Vine Grape) Different Cultivars as a Promising Innovative Cosmetic Raw Material—Phytochemical Profiling, Antioxidant Potential, and Whitening Activity
Source: Molecules. 2023 Sep 29;28(19):6868. doi: 10.3390/molecules28196868 (PMC10574137; doi:10.3390/molecules28196868)
Supplement: Supplementary file 1 [file molecules-28-06868-s001.zip › molecules-2612453-supplementary.pdf]

SUPPLEMENTARY MATERIALS

Shoot Cultures of *Vitis vinifera* (vine grape) Different Cultivars as a Promising Innovative Cosmetic Raw Material – The Phytochemical Profiling, Antioxidant Potential and Whitening Activity

Marta Sharafan[1,2], Magdalena Anna Malinowska[2*], Marta Kubicz[1], Paweł Kubica[1], Marin-Pierre Gémin[3], Cécile Abdallah[3], Manon Ferrier[3], Christophe Hano[4], Nathalie Giglioli-Guivarc'h[3], Elżbieta Sikora[2], Arnaud Lanoue[3], Agnieszka Szopa[1*]

[1] Chair and Department of Pharmaceutical Botany, Medical College, Jagiellonian University, Medyczna 9 St., 30-688 Cracow, Poland; marta.sharafan@doktorant.pk.edu.pl (M.S.); m.kubicz@uj.edu.pl (M.K.); p.kubica@uj.edu.pl (P.K.)

[2] Institute of Organic Chemistry and Technology, Faculty of Chemical Engineering and Technology, Cracow University of Technology, 24 Warszawska St., 31-155 Cracow, Poland; marta.sharafan@doktorant.pk.edu.pl (M.S.); elzbieta.sikora@pk.edu.pl (E.S.)

[3] EA 2106 Biomolécules et Biotechnologies Végétales, UFR des Sciences Pharmaceutiques, Université de Tours, 31 av. Monge, F37200 Tours, France; marin-pierre.gemin@univ-tours.fr (M.-P.G.); cecile.abdallah@ird.fr (C.A.); manon.ferrier@univ-tours.fr (M.F.); (M.F.); nathalie.guivarch@univ-tours.fr (N.G.-G.)

[4] Institut de Chimie Organique et Analytique, Universite d'Orleans-CNRS, UMR 7311 BP 6759, CEDEX 2, 45067 Orleans, France

* Correspondence: a.szopa@uj.edu.pl, tel.: +48-12-620-54-30 (A.S.); magdalena.malinowska@pk.edu.pl, tel.: +48-12-628-25-76 (M.A.M); arnaud.lanoue@univ-tours.fr (A.L.)

Table S1. The quantitative analysis results for the metabolites from shoot cultures of different *V. vinifera* cvs. Values are expressed as mg/g DW (±SD, n=4).

V. vinifera cvs.	Medium variant	m1	m2	m4	m5	m7	m9	m3	m6	m8	m12	m10	m11	m13	m14	m15
		Amino acids						Organic acid	Phenolic acid			Flavan-3-ols				
		L-proline	L-tyrosine	L-isoleucine	L-leucine	L-phenylalanine	L-tryptophan	citric acid	gallic acid	caftaric acid	coutaric acid	Procyanidin B1	Procyanidin B3	catechin	Procyanidin trimer	Procyanidin B4
Johanniter	W1	0.18±0.03	0.33±0.06	0.82±0.16	0.26±0.03	21.38±3.31	0.17±0.03	0.58±0.1	0.53±0.1	0.74±0.12	0.16±0.02	0.42±0.08	0.33±0.05	1.68±0.27	0.21±0.05	0.1±0
	W2	0.18±0.02	0.9±0.14	1.44±0.13	0.34±0.03	50.43±2.33	0.41±0.06	0.55±0.07	0.23±0.04	0.62±0.06	0.16±0.02	1±0.17	0.39±0.07	2.33±0.48	0.41±0.09	0.08±0.02
	W3	0.18±0.03	0.16±0.01	0.36±0.05	0.13±0.03	15.15±1.51	0.12±0.02	1.12±0.13	0.04±0.01	0.78±0.08	0.22±0.04	0.69±0.13	0.49±0.04	2.73±0.31	0.3±0.03	0.11±0.01
	W4	0.2±0.03	0.2±0.02	0.45±0.08	0.19±0.04	20.63±2.9	0.14±0.02	1.34±0.22	0.06±0.01	0.65±0.11	0.19±0.02	0.52±0.02	0.49±0.07	2.55±0.44	0.31±0.04	0.1±0.01
Chardonnay	W1	0.24±0.04	0.52±0.09	1.06±0.25	0.45±0.07	43.42±4.58	0.22±0	0.76±0.07	0.08±0.01	0.39±0.01	0.13±0.01	0.37±0.03	0.3±0.03	1.35±0.17	0.18±0.03	0.1±0.01
	W2	0.3±0.03	0.35±0.07	0.82±0.02	0.16±0.01	27.23±0.97	0.19±0.02	0.67±0.08	1.58±0.09	0.38±0.03	0.08±0.01	0.18±0.01	0.15±0.01	0.9±0.15	0.12±0.02	0.05±0.01
	W3	0.17±0.01	0.26±0.01	0.63±0.09	0.19±0.03	20.14±2.55	0.12±0.02	1.68±0.1	0.04±0.01	0.43±0.07	0.17±0.02	0.38±0.08	0.29±0.02	1.62±0.32	0.15±0.03	0.05±0.01
	W4	0.16±0.02	0.29±0.06	0.41±0.02	0.21±0.03	27.37±4.01	0.16±0.03	1.81±0.34	0.04±0	0.45±0.08	0.15±0.03	0.39±0.02	0.29±0.08	1.42±0.11	0.21±0.01	0.04±0.01
Riesling	W1	0.18±0	0.26±0.04	0.35±0.03	0.32±0.04	24.16±2.62	0.15±0.03	0.41±0.07	0.08±0.01	0.23±0.03	0.06±0.01	0.07±0.01	0.08±0.01	0.29±0.04	0.03±0.01	0.05±0
	W2	0.18±0.02	0.46±0.04	1.03±0.03	0.32±0.07	38.45±5.36	0.29±0.03	0.95±0.08	0.41±0.01	0.25±0.03	0.09±0.02	0.23±0.03	0.32±0.05	1.19±0.16	0.16±0.02	0.07±0.01
	W3	0.17±0.03	0.2±0.04	0.29±0.08	0.11±0.01	11.5±0.92	0.13±0.01	1.03±0.11	0.07±0.01	0.48±0.08	0.18±0.02	0.16±0.02	0.2±0.02	1.1±0.21	0.11±0.01	0.05±0.01
	W4	0.15±0.01	0.09±0.02	0.38±0.05	0.16±0.03	17.19±2.74	0.16±0.02	2.07±0.29	0.05±0.01	0.21±0.02	0.08±0.01	0.06±0.01	0.02±0	0.16±0.01	0.03±0.01	0.01±0
Cabernet cortis	W1	0.27±0.05	0.32±0.07	0.91±0.22	0.45±0.02	61.01±9.02	0.1±0.01	0.7±0.04	0.05±0.01	0.04±0	0.05±0.01	0.1±0.02	0.17±0.02	0.49±0.09	0.05±0.01	0.06±0.01
	W2	0.27±0.05	0.35±0.1	0.82±0.17	0.49±0.1	88.37±13.19	0.12±0.01	0.85±0.08	0.07±0.01	0.06±0.01	0.03±0	0.18±0.05	0.15±0	0.69±0.13	0.07±0.01	0.04±0.01
	W3	0.28±0.02	0.13±0.02	0.7±0.08	0.18±0.01	21.72±2.52	0.12±0.01	1.75±0.25	0.04±0.01	0.2±0.01	0.06±0.01	0.26±0.05	0.22±0.02	1.06±0.13	0.14±0.02	0.07±0.01
	W4	0.41±0.03	0.14±0.01	0.47±0.1	0.16±0.03	27.71±4.5	0.11±0.02	1.96±0.26	0.03±0	0.11±0.01	0.02±0	0.28±0.07	0.25±0.04	1.4±0.27	0.19±0.04	0.14±0.02
Hibernal	W1	0.48±0.06	0.33±0.05	0.86±0.02	0.38±0.06	31.79±3.1	0.12±0.01	1.13±0.17	0.05±0.01	0.15±0.01	0.03±0	0.11±0.02	0.17±0.02	0.62±0.05	0.09±0.01	0.12±0.02
	W2	0.46±0.08	0.34±0.08	0.62±0.12	0.5±0.11	49.57±4.04	0.12±0.02	0.87±0.04	0.09±0.02	0.13±0.02	0.04±0.01	0.33±0.04	0.32±0.05	1.29±0.16	0.15±0.03	0.1±0.01
	W3	0.21±0.03	0.19±0.01	0.4±0.07	0.25±0.04	22.51±2.08	0.1±0.01	1.93±0.36	0.06±0.01	0.51±0.07	0.2±0.03	0.29±0.04	0.22±0.04	1.61±0.18	0.11±0.01	0.03±0.01
	W4	0.27±0.02	0.24±0.05	0.4±0.04	0.17±0.02	28.39±5.26	0.11±0.03	2.31±0.35	0.06±0	0.21±0.05	0.07±0.02	0.42±0.1	0.3±0.07	1.81±0.23	0.15±0.02	0.03±0.01
Regent	W1	0.17±0.02	0.33±0.07	0.45±0.11	0.52±0.08	51.42±9.02	0.14±0.02	0.76±0.11	0.05±0.01	0.31±0	0.09±0.01	0.24±0	0.27±0.04	1.07±0.14	0.12±0.03	0.04±0.01
	W2	0.19±0.04	0.32±0.04	0.57±0.01	0.66±0.18	43.21±3.63	0.09±0	1.79±0.28	0.04±0.01	0.1±0.02	0.04±0	0.19±0.02	0.26±0.02	1.1±0.05	0.1±0.01	0.03±0
	W3	0.16±0	0.26±0.01	0.92±0.04	0.27±0.07	31.02±0.19	0.23±0.02	1.6±0.35	0.06±0.01	0.49±0.13	0.13±0.04	0.66±0.2	0.36±0	2.33±0.28	0.21±0.01	0.05±0
	W4	0.22±0.01	0.32±0.03	0.81±0.02	0.42±0.05	37.88±3.26	0.16±0.02	1.73±0.33	0.03±0	0.13±0.03	0.04±0.01	0.37±0.05	0.39±0.04	2.19±0.17	0.18±0.05	0.06±0.01
Solaris	W1	0.63±0.07	0.27±0.02	0.54±0.09	0.47±0.03	45.3±8.76	0.11±0.02	1.03±0.19	0.05±0.01	0.06±0.01	0.01±0	0.15±0.02	0.13±0.01	0.75±0.06	0.07±0.01	0.09±0.01
	W2	0.52±0.1	0.41±0.07	1.05±0.23	0.62±0.06	80.98±8.42	0.23±0.03	1.41±0.33	0.09±0	0.23±0.04	0.04±0.01	0.19±0.04	0.24±0.03	0.87±0.18	0.07±0.02	0.18±0.01
	W3	1.36±0.11	0.22±0.01	0.41±0.07	0.11±0.02	22.82±0.29	0.03±0	1.89±0.3	0.04±0	0.06±0.01	0.02±0	0.22±0.03	0.21±0.05	1.02±0.09	0.08±0	0.13±0.03
	W4	0.25±0.04	0.11±0.01	0.51±0.09	0.27±0.03	28.04±5.15	0.07±0.01	3.02±0.43	0.06±0.01	0.1±0.02	0.01±0	0.16±0.02	0.18±0.01	1.08±0.12	0.11±0.01	0.03±0.01

Table S1 *continued*

V. vinif.era cvs.	Medium variant	m16	m17	m19	m20	m21	m22	m25	m29	m18	m26	m27	m24	m32	m35	m23
		Flavan-3-ols								Flavonols			Stilbenoids DP1			DP2
		procyanidin B2	epicatechin	procyanidin C1	procyanidin gallate 1	procyanidin gallate	procyanidin dimer 5	catechin gallate	procyanidin gallate 3	quercetine glucoside	quercetin glucuronide	quercetin-3-O-galactoside	piceid	trans resveratrol	cis resveratrol	restrytisol 1
Johanniter	W1	0.14±0.04	1.25±0.21	0.14±0.04	0.09±0.01	0.97±0.08	0.11±0.02	1.55±0.26	0.04±0.01	0.03±0	0.23±0.04	0.05±0.01	1.96±0.36	0.16±0.04	0.05±0	0.02±0
	W2	0.24±0.02	1±0.14	0.2±0.02	0.1±0.02	1.44±0.36	0.19±0.05	2.62±0.13	0.06±0.01	0.03±0	0.25±0.03	0.07±0.01	2.47±0.27	0.05±0.01	0.02±0.01	0.04±0.01
	W3	0.18±0.02	1.13±0.08	0.16±0.03	0.08±0.01	0.76±0.13	0.17±0.03	1.08±0.08	0.04±0	0.05±0.01	0.18±0.01	0.04±0.01	1.38±0.2	0.02±0	0.01±0.01	0.03±0
	W4	0.15±0.02	1.11±0.11	0.18±0.04	0.08±0.01	0.66±0.04	0.14±0.02	1.21±0.12	0.04±0	0.06±0.01	0.11±0.01	0.06±0.01	2.39±0.36	0.03±0	0±0.01	0.05±0
Chardonnay	W1	0.14±0.02	0.73±0.13	0.21±0.03	0.09±0.02	0.69±0.08	0.08±0.01	1.16±0.18	0.04±0	0.02±0	0.08±0	0.05±0.01	1.4±0.32	0.14±0.03	0.02±0.03	0.05±0.01
	W2	0.11±0.03	0.81±0.16	0.1±0.01	0.04±0.01	0.4±0.09	0.05±0.01	0.74±0.17	0.03±0	0.01±0	0.1±0.02	0.05±0.01	1.2±0.22	0.14±0.02	0.01±0	0.03±0
	W3	0.09±0.01	0.57±0.06	0.07±0.01	0.04±0.01	0.3±0.05	0.07±0.01	0.54±0.1	0.02±0	0.02±0	0.06±0.01	0.04±0.01	0.84±0.1	0.02±0	0.01±0.01	0.03±0.01
	W4	0.06±0.01	0.35±0.05	0.05±0.01	0.05±0	0.29±0.08	0.09±0.01	0.55±0.07	0.02±0.01	0.02±0	0.1±0.01	0.05±0.01	0.58±0.08	0.02±0	0±0.01	0.04±0.01
Riesling	W1	0.05±0.01	0.49±0.07	0.03±0.01	0.04±0.01	0.25±0.04	0.02±0	0.58±0.04	0.01±0	0.13±0.01	0.04±0.01	0.02±0	0.96±0.14	0.24±0.03	0.02±0	0.07±0.01
	W2	0.1±0.03	0.8±0.1	0.13±0.02	0.1±0.01	0.65±0.07	0.06±0	1.5±0.18	0.04±0.01	0.04±0	0.1±0.02	0.04±0.01	3.04±0.27	0.09±0.01	0.01±0	0.05±0.01
	W3	0.07±0.01	0.31±0.01	0.07±0.01	0.04±0	0.28±0.01	0.07±0.01	0.58±0.06	0.01±0	0.1±0.02	0.14±0.02	0.04±0.01	1.17±0.11	0.03±0	0±0.01	0.02±0
	W4	0.02±0	0.07±0.01	0.03±0	0.02±0	0.11±0.03	0.01±0	0.16±0.03	0.01±0	0.04±0.01	0.12±0.02	0.03±0.01	0.45±0.02	0.06±0.01	0.01±0	0.05±0.01
Cabernet cortis	W1	0.06±0.01	0.44±0.09	0.1±0.02	0.1±0.01	0.44±0.05	0.06±0.02	1.51±0.17	0.03±0.01	0.02±0	0.02±0	0.05±0.01	0.66±0.1	0.02±0	0.01±0	0.02±0
	W2	0.05±0.01	0.32±0.09	0.04±0.01	0.07±0.01	0.49±0.01	0.03±0.01	1.43±0.17	0.03±0.01	0.01±0	0.02±0	0.03±0.01	0.78±0.22	0.01±0	0.01±0	0.02±0
	W3	0.08±0.02	0.97±0.13	0.07±0.01	0.11±0.01	0.78±0.08	0.08±0.01	1.91±0.08	0.03±0	0.02±0	0.05±0	0.03±0.01	0.34±0.05	0.01±0	0±0.01	0.03±0
	W4	0.12±0.02	1.92±0.14	0.12±0.02	0.16±0.04	0.97±0.17	0.14±0.02	2.59±0.46	0.05±0.01	0.03±0	0.04±0.01	0.05±0.01	0.89±0.13	0.03±0.01	0±0	0.07±0.01
Hibernal	W1	0.14±0.02	0.87±0.09	0.1±0.02	0.17±0.03	1.14±0.14	0.05±0.01	2.75±0.14	0.07±0.01	0.03±0.01	0.06±0.01	0.09±0.02	1.04±0.12	0.08±0.01	0.01±0.01	0.03±0
	W2	0.13±0.01	1.1±0.19	0.17±0	0.21±0.04	1.22±0.15	0.09±0.01	3.09±0.39	0.07±0.01	0.02±0	0.07±0.01	0.09±0	2.59±0.47	0.04±0.01	0±0.01	0.03±0
	W3	0.06±0.02	0.45±0.08	0.05±0.01	0.06±0.01	0.55±0.08	0.07±0.01	1.9±0.14	0.04±0	0.05±0	0.08±0.01	0.03±0.01	1.13±0.22	0.04±0.01	0.02±0.01	0.05±0.01
	W4	0.07±0.01	0.59±0.08	0.1±0.03	0.07±0.01	0.54±0.18	0.11±0.01	1.71±0.33	0.03±0.01	0.05±0.01	0.06±0.01	0.05±0.01	2.63±0.69	0.03±0.01	0.01±0.01	0.06±0
Regent	W1	0.05±0.01	0.73±0.13	0.06±0.01	0.11±0.01	0.75±0.22	0.08±0.02	1.78±0.09	0.03±0	0.07±0.01	0.05±0.01	0.02±0	1.48±0.31	0.07±0.01	0.01±0.01	0.04±0.01
	W2	0.03±0	0.31±0.06	0.03±0	0.06±0.01	0.35±0.04	0.06±0.01	0.99±0.04	0.01±0	0.04±0.01	0.02±0	0.01±0	1.39±0.15	0.1±0.01	0.05±0.04	0.11±0.01
	W3	0.05±0.02	0.78±0.13	0.05±0.01	0.1±0	0.6±0.03	0.13±0.02	1.8±0.48	0.05±0.01	0.07±0.02	0.12±0	0.04±0	1.14±0.31	0.05±0.01	0.01±0	0.08±0.02
	W4	0.09±0.02	0.64±0.09	0.12±0.02	0.11±0.02	0.95±0.25	0.12±0	2.2±0.19	0.04±0	0.02±0	0.05±0	0.04±0	1.78±0.36	0.04±0.01	0.01±0	0.09±0.01
Solaris	W1	0.07±0.01	0.49±0.08	0.08±0.01	0.17±0.02	0.81±0.05	0.04±0.01	2.11±0.34	0.05±0	0.01±0	0.03±0	0.06±0.01	1.39±0.17	0.03±0	0.01±0	0.02±0
	W2	0.19±0.02	1.18±0.08	0.23±0.06	0.26±0.05	1.16±0.1	0.05±0	2.6±0.33	0.09±0	0.02±0	0.13±0.01	0.1±0.02	2.38±0.47	0.04±0	0.01±0	0.02±0
	W3	0.14±0.03	0.97±0.08	0.1±0.01	0.21±0	1.13±0.16	0.1±0.02	1.91±0.38	0.04±0	0.01±0	0.04±0.01	0.12±0	1.46±0.25	0.01±0	0.01±0.01	0.04±0.01
	W4	0.04±0	0.4±0.11	0.03±0	0.08±0.02	0.31±0.02	0.07±0.01	1.61±0.25	0.03±0.01	0.01±0	0.09±0.01	0.04±0	0.62±0.12	0.02±0	0.01±0.01	0.04±0

Table S1 *continued*

V. vinifera cvs.	Medium variant	m28	m31	m37	m38	m41	m42	m45	m36	m43	m33	m34	m39	m40	m44	m46
		Stilbenoid DP2							Stilbenoid DP3		Stilbenoid DP4					
		ampelopsin A	resveratrol dimer glycosylé	Z-ε-viniferin	E-ε-viniferin	α-viniferin	ω-viniferin	δ-viniferin	resveratrol trimer 1	resveratrol trimer 4	hopeaphenol	isohopeaphenol	resveratrol tetramer 3	resveratrol tetramer 4	vitisin B	resveratrol tetramer 6
Johanniter	W1	0.08±0.01	1.59±0.13	0.08±0.02	0.24±0.05	0.02±0	1.51±0.22	0.02±0.01	0.41±0.01	0.08±0.02	0.1±0	0.16±0.01	0.01±0	0.05±0.01	0.05±0	0.01±0
	W2	0.05±0.01	3.79±0.12	0.05±0.01	0.17±0.04	0.01±0	1.7±0.1	0.02±0.01	0.47±0.11	0.07±0.01	0.13±0.01	0.11±0.01	0.01±0	0.09±0.01	0.03±0	0±0
	W3	0.33±0.05	2.1±0.04	0.08±0.01	0.56±0.06	0.06±0	1.37±0.15	0.01±0	0.52±0.07	0.11±0.01	0.49±0.06	0.2±0.04	0.01±0.01	0.05±0	0.6±0	0.03±0
	W4	0.11±0.02	2.39±0.36	0.06±0.01	0.33±0.06	0.04±0	3.08±0.24	0.04±0.02	0.63±0.08	0.13±0.01	0.18±0.02	0.18±0.01	0.01±0	0.07±0	0.22±0.06	0.02±0
Chardonnay	W1	0.06±0.01	1.66±0.23	0.1±0.02	1.26±0.04	0.08±0	3.06±0.05	0.08±0.01	0.26±0.04	0.36±0.01	0.07±0.01	0.35±0.01	0.03±0.01	0.02±0	0.03±0	0.02±0
	W2	0.03±0.01	0.97±0.09	0.02±0.02	0.08±0.01	0.01±0	0.99±0.22	0.01±0.01	0.21±0.04	0.11±0.02	0.01±0.01	0.04±0	0±0	0.02±0	0.01±0	0±0
	W3	0.09±0.01	1.57±0.09	0.13±0.04	0.35±0.05	0.04±0.01	2.82±0.15	0.04±0.02	0.32±0.04	0.38±0.03	0.06±0.01	0.28±0.03	0.02±0	0.02±0	0.1±0.02	0.02±0
	W4	0.14±0	1.39±0.22	0.11±0.02	0.27±0.03	0.03±0	1.52±0.07	0.03±0.03	0.25±0.03	0.25±0.01	0.1±0.01	0.17±0.02	0.02±0	0.01±0	0.17±0.01	0.02±0
Riesling	W1	0.15±0.02	2.29±0.19	0.3±0.01	1.64±0.15	0.13±0.02	10.45±0.27	0.03±0	0.36±0.05	0.7±0.1	0.65±0.08	0.64±0.08	0.07±0.01	0.03±0	0.24±0.04	0.05±0
	W2	0.03±0.01	1.54±0.14	0.05±0.01	0.26±0.05	0.05±0	2.01±0.39	0.01±0.01	0.43±0.06	0.13±0.01	0.08±0.01	0.14±0.03	0.01±0	0.06±0.01	0.02±0	0.01±0
	W3	0.18±0.02	1.17±0.1	0.08±0	0.39±0.08	0.03±0	1.14±0.19	0.01±0	0.15±0.02	0.17±0.05	0.5±0.07	0.14±0.02	0.01±0	0.01±0	0.29±0.02	0.01±0
	W4	0.08±0	0.67±0.12	0.08±0.02	0.19±0.02	0.01±0	1.59±0.12	0.01±0	0.32±0.05	0.13±0.02	0.26±0.06	0.09±0.01	0.01±0	0.02±0	0.17±0.02	0.01±0
Cabernet cortis	W1	0.01±0	1.86±0.22	0.04±0	0.18±0.01	0.01±0	3.44±0.04	0.03±0.01	0.43±0.03	0.19±0.04	0.03±0.01	0.07±0.02	0.01±0	0.06±0.01	0.06±0.01	0.01±0
	W2	0±0	0.95±0.17	0.02±0.02	0.05±0.02	0.01±0	3.27±0	0.01±0	0.33±0.04	0.13±0.07	0.01±0	0.05±0.02	0±0	0.08±0.01	0.01±0	0±0
	W3	0.03±0	1.76±0.3	0.06±0.01	0.45±0.05	0.04±0.01	2.19±0.24	0.02±0	0.29±0.03	0.17±0.01	0.04±0	0.08±0.02	0.02±0	0.04±0	0.48±0.06	0.02±0
	W4	0.01±0	3.27±0.68	0.08±0	0.41±0.09	0.08±0.02	3.17±0.06	0.02±0	0.68±0.13	0.41±0.1	0.04±0	0.29±0.09	0.03±0	0.06±0.01	0.22±0	0.02±0
Hibernal	W1	0.01±0	1.44±0.3	0.03±0	0.3±0.03	0.02±0.01	4.61±0.25	0.03±0.01	0.54±0.06	0.25±0.02	0.02±0	0.09±0.01	0.01±0	0.09±0.02	0.02±0	0.01±0
	W2	0±0	1.81±0.04	0.08±0.01	0.15±0.02	0.02±0	3.32±0.24	0.01±0	0.48±0.08	0.14±0.01	0.01±0	0.14±0.03	0.01±0	0.08±0.01	0.01±0	0.01±0
	W3	0.03±0	2.62±0.15	0.11±0	0.59±0.15	0.09±0.01	5.69±0.14	0.02±0	0.77±0.12	0.67±0.04	0.1±0.01	0.45±0.05	0.03±0	0.07±0.01	0.09±0.01	0.03±0
	W4	0.02±0	2.26±0.38	0.09±0.02	0.23±0.03	0.02±0	3.82±0.6	0.03±0	0.69±0.14	0.38±0.08	0.08±0.02	0.19±0.03	0.02±0	0.05±0	0.07±0.01	0.02±0
Regent	W1	0.04±0	2.38±0.17	0.28±0.01	0.39±0.03	0.05±0	3.2±0.21	0.07±0.03	0.44±0.08	0.39±0.04	0.14±0.03	0.72±0.06	0.05±0	0.04±0.01	0.05±0.01	0.03±0
	W2	0.03±0.01	3.51±0.14	0.29±0.04	0.47±0.08	0.06±0	11.28±0.36	0.05±0.01	0.74±0.15	0.99±0.14	0.12±0.01	0.83±0.07	0.05±0	0.09±0.02	0.08±0.01	0.03±0
	W3	0.21±0.05	1.63±0.15	0.27±0.05	0.56±0.07	0.06±0.01	2.89±0.59	0.03±0	0.75±0.09	0.13±0.03	0.42±0	0.57±0.01	0.05±0.01	0.04±0.01	0.34±0.06	0.04±0
	W4	0.12±0.02	2.36±0.3	0.26±0.02	0.44±0.09	0.05±0	6.63±0.31	0.02±0	1.09±0.06	0.39±0.03	0.22±0.01	0.47±0.02	0.05±0	0.08±0	0.28±0.01	0.03±0
Solaris	W1	0.01±0	2.28±0.29	0.03±0	0.11±0.01	0.01±0	1.06±0.15	0.02±0.02	0.21±0.03	0.24±0.02	0.03±0	0.15±0.01	0.01±0	0.04±0	0.01±0	0.01±0
	W2	0±0	1.76±0.24	0±0	0.05±0	0±0	1.9±0.43	0.02±0.02	0.21±0.06	0.13±0.02	0.01±0	0.05±0.01	0±0	0.04±0	0.01±0	0±0
	W3	0.01±0	1.38±0.07	0.09±0	0.12±0.01	0.01±0	1.64±0.13	0.01±0	0.29±0.01	0.26±0.03	0.03±0.01	0.23±0.04	0.02±0	0.09±0.02	0.01±0	0.01±0
	W4	0.01±0	0.78±0.05	0.11±0.02	0.27±0.06	0.02±0	2.87±0.13	0.02±0	0.36±0.11	0.23±0.02	0.07±0.02	0.29±0.05	0.04±0	0.08±0.01	0.06±0.01	0.01±0
